# Lime/Sodium Carbonate Treated Seawater to Improve Flocculation and Sedimentation of Clay-Based Tailings

**DOI:** 10.3390/polym13234108

**Published:** 2021-11-25

**Authors:** Francisco Pulgar, Luis Ayala, Matías Jeldres, Pedro Robles, Pedro G. Toledo, Iván Salazar, Ricardo I. Jeldres

**Affiliations:** 1 Departamento de Ingeniería Química y Procesos de Minerales, Facultad de Ingeniería, Universidad de Antofagasta, P.O. Box 170, Antofagasta 1240000, Chile; francisco.pulgar.gonzalez@ua.cl (F.P.); hugo.jeldres.valenzuela@ua.cl (M.J.); 2 Faculty of Engineering and Architecture, Universidad Arturo Prat, Almirante Juan José Latorre 2901, Antofagasta 1240000, Chile; luisayala01@unap.cl; 3 Escuela de Ingeniería Química, Pontificia Universidad Católica de Valparaíso, Valparaíso 2340000, Chile; pedro.robles@pucv.cl; 4 Departamento de Ingeniería Química, Universidad de Concepción, Concepción 4030000, Chile; 5 Departamento de Ingeniería Civil, Universidad Católica del Norte, Antofagasta 1270709, Chile; isalazar@ucn.cl

**Keywords:** tailings flocculation, seawater, calcium and magnesium removal, lime, sodium carbonate

## Abstract

Seawater treated with lime and sodium carbonate in different proportions to reduce magnesium and calcium contents is used in flocculation and sedimentation tests of artificial quartz and kaolin tailings. Solid complexes were separated from water by vacuum filtration, and factors such as lime/sodium carbonate ratio, kaolin content, flocculation time, and flocculant dose are evaluated. The growth of the aggregates was captured in situ by a focused beam reflectance measurement (FBRM) probe. Solid magnesium and calcium complexes are formed in raw seawater at pH 11, impairing the performance of flocculant polymers based on polyacrylamides. The results show that the settling rate improved when the treatment’s lime/sodium carbonate ratio increased. That is, when a greater removal of magnesium is prioritized over calcium. The amount of magnesium required to be removed depends on the mineralogy of the system: more clay will require more significant removal of magnesium. These results respond to the structural changes of the flocs, achieving that the more magnesium is removed, the greater the size and density of the aggregates. In contrast, calcium removal does not significantly influence flocculant performance. The study suggests the necessary conditions for each type of tailing to maximize water recovery, contributing to the effective closure of the water cycle in processes that use seawater with magnesium control.

## 1. Introduction

An important number of mineral deposits are located in arid or semi-arid regions, where any water consumption has significant economic and socio-environmental repercussions [1,2]. Thus, reducing water consumption in the concentration stages is an urgent task [3,4]. In this sense, solid–liquid separation has a fundamental role because it is the stage in which the greatest amount of water is recovered [5]. This process separates solid particles by flocculating them in water and settling by gravity [6,7]. As a result, a highly concentrated sediment is obtained that is transported through a pipe to a disposal area for further dewatering and consolidation. At the same time, the clarified overflow is recycled in the upstream operations. A common flocculant is a high molecular weight anionic polyacrylamide (A-PAM), which generates high sedimentation rates when applied at relatively low doses [8,9,10].

Currently, several mining operations use seawater in their processes, either directly or after desalination by reverse osmosis [2,11]. For example, by 2030, the consumption of seawater in Chile would increase by 156% compared to 2019, which represents 47% of the water required by the copper mining at the national level [2]. The use of desalinated seawater ensures an adequate water supply. However, desalination plants pose numerous environmental challenges, mainly related to the wastewater concentrated in salt and chemicals. Discarding can affect coastal water quality and dramatically change the marine environment [12,13]. Furthermore, this process requires energy supplied by fuels, which implies high emissions of atmospheric pollutants [14]. The direct use of seawater offers the advantage of avoiding the construction of desalination plants, reducing the associated economic and environmental costs. However, this implementation should be directed to new plants since older facilities are not prepared to the withstand high salinities [2].

Several studies have been dedicated to evaluating the consequences of a saline environment in the mineral flocculation processes and its impact on the thickening stages. For example, Ji et al. [15] used different flocculants to sediment quartz and albite in a highly saline solution, finding high sedimentation rates and a clearer supernatant. Liu et al. [16] suggested that salts enhance the aggregation of bentonite and illite, but hinder the aggregation of kaolinite. Recently, Jeldres et al. [17] and Quezada et al. [18] have used NaCl brines, showing that a saline medium can favor the flocculation of mineral particles by increasing the adsorption of the flocculant on the surface of the particles. These results are interesting because they challenge the idea that flocculants roll up in the presence of salts, losing the adsorptive capacity of their mineral particles. Thus, salinity can favor or harm flocculation, depending on the type of flocculant and mineral considered. The process is more complicated in seawater, especially when the concentration circuit is carried out in alkaline conditions that are characteristic of froth flotation operations. It is typical for the copper minerals processing to operate at a pH higher than 10.5, with the aim of depressing non-valuable minerals such as pyrite, which tend to float due to their hydrophobic nature, thus contaminating the concentrate [19,20]. However, this strategy cannot be implemented when using seawater since solid Ca/Mg complexes can arise, producing a buffering effect at a pH above 10 and a considerable reduction in the recovery of molybdenite [21,22]. Furthermore, the consequences for thickening operations are challenging. The few studies that have been performed in this regard show that the presence of precipitates leads to considerably lower sedimentation rates. For example, Ramos et al. [23] analyzed the flocculation of mine tailings using seawater in a wide range of pH in the pulp, and reported a severe detriment to the sedimentation rate once the pH of the slurry reaches the value at which solid precipitates (pH > 10.3). In their main results, Ramos et al. found that magnesium causes the greatest detriment. They also found that calcium does not affect the process within the pH range considered (pH < 11.1). Ramos et al. suggested that magnesium precipitates would have a higher affinity for the flocculant, which impairs the unique interaction of the polymer with the mineral. This forced the use of high doses, first to saturate the surface of the precipitates, and then to flocculate the mineral particles.

To reduce the impact caused by precipitates and solid complexes on foam flotation, Castro [24] proposed reducing the magnesium content of seawater by treating it with lime. Later, Jeldres et al. [25] supplemented the strategy by using a mixture of lime and sodium carbonate to reduce magnesium and calcium content. The authors achieved good recovery of molybdenite and chalcopyrite at an alkaline pH, promoting pyrite depression. Recently, Jeldres et al. [26] studied thickening operations, finding that a lime removal treatment can improve the performance of the thickeners. The authors considerably increased the sedimentation rate of mineral tailings at pH 11, obtaining higher values than at natural pH. More recently, Arias et al. [27] proposed a biotechnological treatment to remove divalent ions from seawater, using a fluidized bed bioreactor completed with the halotolerant ureolytic strain Bacillus subtilis LN8B. These latest studies [26,27] have yielded promising results in reducing magnesium in seawater, opening up a new line of research that needs to be explored systematically.

In this work, different mixtures of lime with sodium carbonate are used to produce seawater with varying magnesium contents. These treated waters are evaluated in flocculation and sedimentation tests of synthetic quartz and kaolin tailings in terms of lime/sodium carbonate ratio, kaolin content, flocculation time, and flocculant dose.

## 2. Materials and Methods

### 2.1. Materials

Seawater from San Jorge Bay in Antofagasta (Chile) was filtered through a U.V. purification system to eliminate bacterial activity. The electrical conductivity was 50.2 mS/cm at 25 °C, and the pH was natural (pH 7.5). The concentration of the primary ions was determined by the methods indicated in Table 1.

**Figure 1:**
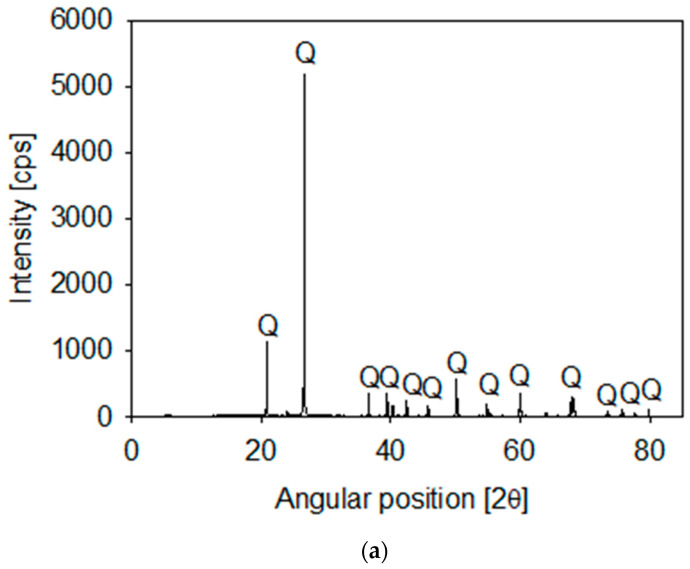

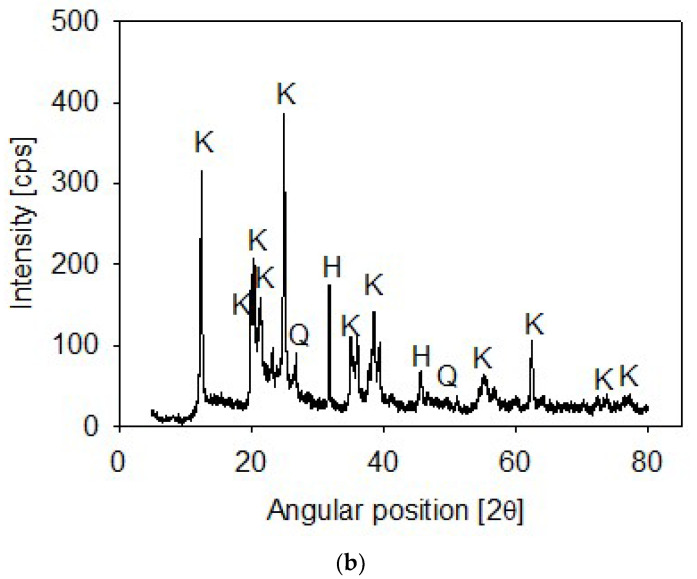
X-ray diffraction of quartz (**a**) and kaolin (**b**) powder, showing kaolinite (K), halite (H) and quartz (Q).

Synthetic tailings were prepared from mixtures of quartz and kaolin in different proportions. The quartz particles were available in our lab (XRD in Figure 1a) and the kaolin was procured from Ward’s Science (Rochester, NY, USA). The kaolin composition included mainly kaolinite and halite and a small proportion of SiO_2_ (XRD in Figure 1b). The XRD analyses were performed on a Bruker D8 Advance X-ray diffractometer (Bruker, Karlsruhe, Germany). Once collected, diffraction data were processed and analyzed using the latest versions of the International Centre for Diffraction (ICDD) databases.

**Figure 2:**
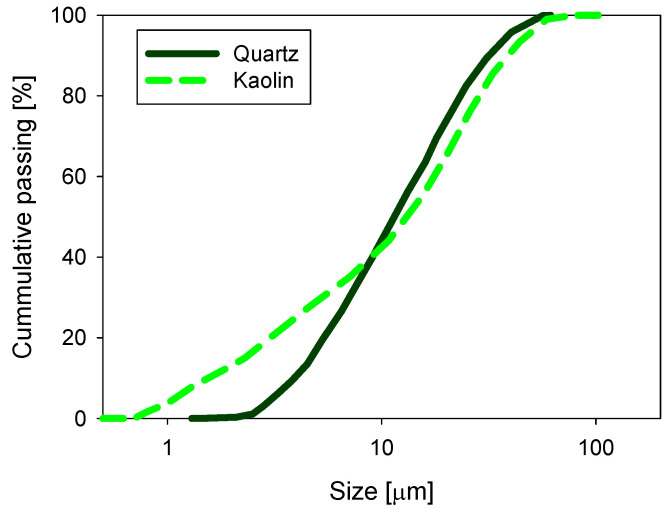
Size distribution for quartz and kaolin particles in distilled water at natural pH.

Volume-weighted particle size distribution (PSD) was measured by laser diffraction using a Microtrac S3500 instrument (Verder Scientific, Newtown, PA, USA). As shown in Figure 2, 10% of the particles were smaller than d10 = 1.8 and 3.8 µm in the kaolin and quartz samples, respectively. SNF 704, provided by SNF Chile S.A., was used as a flocculant. This reagent has a molecular weight of 18 × 10^6^ and a medium charge density (30–50% anionic functionalities). The alkalizing agents used to precipitate magnesium at alkaline pH were analytical-grade lime and sodium carbonate, obtained from Sigma-Aldrich (Santiago, Chile). The pH of the suspensions was controlled with sodium hydroxide, obtained from Sigma-Aldrich, Chile.

### 2.2. Magnesium Removal Treatment

Magnesium ions were partially removed from seawater by precipitation with lime and sodium carbonate in different proportions and overall concentration of 0.07 M. The resulting solution was mixed for 30 min at room temperature, generating a highly alkaline environment that favored the formation of the calcium and magnesium precipitates that were finally separated by vacuum filtration. This filtrate, referred to as treated seawater throughout this document, was used in flocculation and sedimentation tests of artificial quartz and kaolin tailings. Ca and Mg concentrations were measured by inductively coupled plasma mass spectrometry (ICP-MS, Varian 220 FS Atomic Absorption Spectrophotometer, Varian, Palo Alto, CA, USA).

### 2.3. Flocculation Kinetic

In a 1 L beaker, 24 g of the quartz–kaolin-based mineral was mixed with 246 g of water, and the pH was adjusted to 11 with sodium hydroxide. For this, a stirring rate (600 rpm) was applied for 5 min using a 30 mm diameter turbine impeller at the end of a vertical axis (4 mm diameter) placed 20 mm above the bottom of the container. Subsequently, the stirring was reduced to 220 rpm, then the flocculant solution and the remaining volume of water were added to reach 300 g of suspension. This methodology ensured the same solids concentration (8% by weight) for all the flocculation experiments. The calcium and magnesium that were not precipitated in the lime/sodium carbonate treatment did so during the pH control with sodium hydroxide in the conditioning for the flocculation tests.

The evolution of the aggregate size of each pulp was determined using the focused beam reflectance measurement (FBRM) system (Particle Track E25, Mettler Toledo, Columbus, OH, USA), which consists of a processing unit and a probe with a 19 mm diameter tip and a sapphire window (14 mm diameter) at the measuring tip. The probe was inserted vertically into the container with the pulp, 10mm above the stirrer and 20 mm off-axis. The FBRM probe features a laser that is focused through the sapphire window and scans a circular path at a tangential velocity of 2 m/s. Once the beam encounters suspended solids in the focal plane, backscattered light is generated. A chord length is obtained from the persistence of any high backscattered light intensity and the speed of the laser. The software processes the recorded data into histograms of the counts corresponding to chord lengths in selected channel sizes ranging from 1 μm to 1 mm as quickly as every 2 s. In this case, the chord length distributions (CLD) represent 100 channels in the full range, but the histograms are presented as line graphs for easy comparison. The FBRM system offers two types of particle size distributions, the unweighted CLD, which is more sensitive to finer particles, and the squared-weighted CLD, which is more susceptible to coarse particle aggregates. The raw data were processed in this work without any weighing to detect dispersed fine particles. However, the average size was obtained as a function of the square-weighted size distribution.

### 2.4. Sedimentation Tests

The settling rate was determined by interrupting the flocculation tests at specific preset times of 20, 40, 60, and 80 s. Then, the suspension was poured from the bottom of the flocculation cell into a cylindrical tube (35 mm inner diameter). The cylinder, with its contents, was slowly inverted three times and then placed on a surface to determine the sedimentation rate classically.

### 2.5. Fractal Dimension

The fractal dimension of the aggregates was determined according to the methodology of Heath et al. [28], expressed in the equation

(1) $$U_{h}=\frac{\bar{d_{agg}^{2}}g\left(\rho_{s}-\rho_{l}\right){\left(\frac{\overset{↼}{d_{agg}}}{\overset{↼}{d_{p}}}\right)}^{D_{f}-3}}{18𝜇}\;\;\;{\left(1-\phi_{s}{\left(\frac{\overset{↼}{d_{agg}}}{\overset{↼}{d_{p}}}\right)}^{3-D_{f}}\right)}^{4.65}$$

where $U_{h\;}$ is the hindered settling rate in m/s, *d_p_* is the average size of the primitive particles, *d_agg_* is the average size of the aggregates after some flocculation time, 𝜌*_s_* and 𝜌*_l_* are, respectively, the densities of the solid and liquid phases, *g* is the acceleration of gravity, 𝜇 is the fluid viscosity, $\phi_{s}$ is the solid fraction, and *D_f_* is the mass fractal dimension. To determine the fractal dimension, the hindered settling rate of the previous section was used, and the squared weighted mean chord length was used for the average size of the aggregates. All the other parameters in the equation were constant for all the systems studied.

## 3. Results and Discussion

Water recovery in mineral concentration processes using seawater requires fine control of magnesium. In this study, seawater was treated with lime and sodium carbonate to precipitate magnesium, and then filtered. Various proportions of lime and sodium carbonate were used. Treated seawater was used in flocculation and sedimentation tests of artificial tailings of quartz and kaolin to evaluate the lime/sodium carbonate ratio, kaolin content, flocculation time, and flocculant dose. The growth of the aggregates was captured by the FBRM probe.

### 3.1. Seawater Treatment

The main reactions when seawater is treated with various proportions of lime and sodium carbonate are as follows.

(2) $$Ca{\left(OH\right)}_{2}+Mg^{2+}⟷Mg{\left(OH\right)}_{2}+Ca^{2+}$$

(3) $$Ca^{2+}+2OH^{-}⟷\;Ca{\left(OH\right)}_{2}$$

(4) $$Ca{\left(OH\right)}_{2}+SO_{4}^{2-}+2H_{2}O⟷CaSO_{4}\cdot 2H_{2}O+2OH^{-}$$

(5) $$Mg{\left(OH\right)}_{2}+SO_{4}^{2-}⟷\;MgSO_{4}+2OH^{-}$$

(6) $$Ca{\left(OH\right)}_{2}+CO_{3}^{2-}⟷CaCO_{3}+2OH^{-}$$

(7) $$Mg{\left(OH\right)}_{2}+CO_{3}^{2-}⟷MgCO_{3}+2OH^{-}$$

Magnesium combines to give rise to various complexes with varying solubilities. The solubility constants are summarized in Table 2.

**Figure 3:**
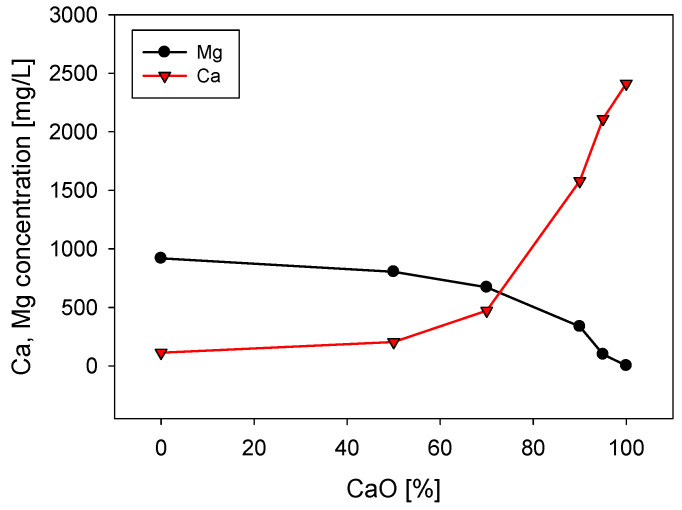
Calcium and magnesium concentrations in seawater treated with different lime/sodium carbonate ratios with a concentration of 0.07 M. Initial Mg and Ca are, respectively, 1420 mg/L and 420 mg/L.

Figure 3 shows the concentration of calcium and magnesium in seawater after being treated with different proportions of lime and sodium carbonate, always maintaining a total concentration of 0.07 M of these reagents. When using 100% sodium carbonate, the magnesium concentration decreases from 1420 to 920 mg/L because it precipitates as magnesium carbonate. However, as the lime/sodium carbonate ratio increases, the amount of magnesium in the solution decreases significantly, with almost complete removal when 100% lime is used. This result shows that lime precipitates more magnesium as magnesium hydroxide than sodium carbonate as magnesium carbonate. The explanation lies in the higher solubility of magnesium carbonate, 2.61 × 10^−3^ at 25 °C, versus the solubility of magnesium hydroxide, which is only 1.77 × 10^−4^ at 25 °C. Regarding calcium, the concentration decreases from 400 to 113 mg/L when using 100% sodium carbonate and increases when the lime content increases, reaching 2412 mg/L when using 100% lime. Seawater treatment with lime/sodium carbonate ratios below 50% is ineffective for magnesium reduction, as shown in Figure 3.

### 3.2. Implications of Magnesium Content

**Figure 4:**
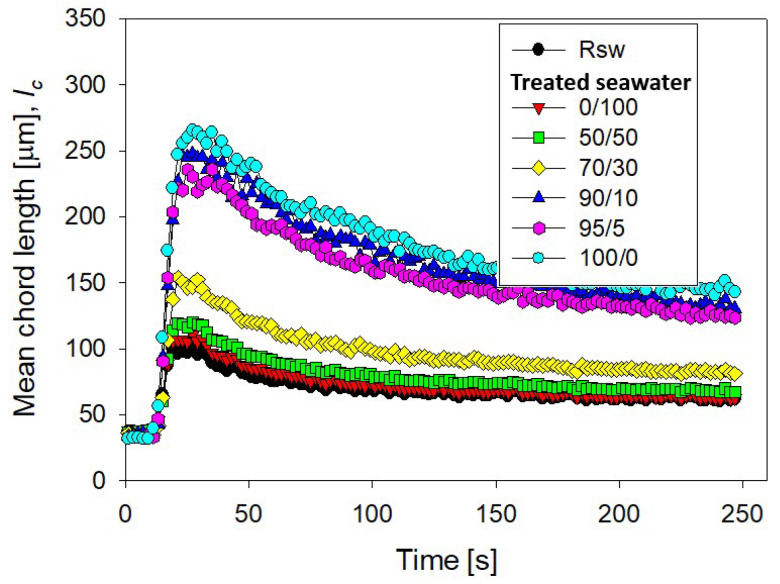
Clay-based tailing flocculation kinetics in raw seawater (Rsw) and seawater treated with different ratios of lime/sodium carbonate. Quartz/kaolin = 80/20, pH 11, stirring rate 220 rpm, and flocculant dose 17 g/t.

**Figure 5:**
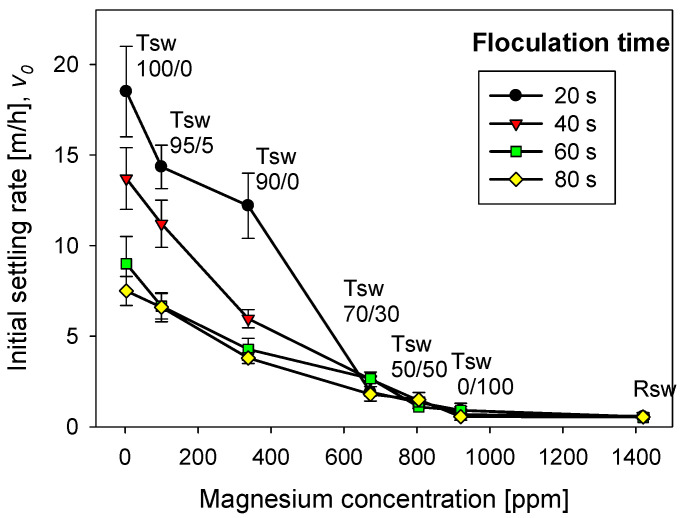
Effect of seawater treatment and flocculation time on the settling rate of flocculated tailings. Quartz/kaolin = 80/20, pH 11, stirring rate 220 rpm, and flocculant dose 17 g/t. Seawater treated with lime/sodium carbonate in different ratios (Tsw). Raw seawater is included for comparison.

The magnesium content in tailings prepared with seawater treated with different lime/sodium carbonate ratios has great implications for flocculation kinetics and settling rates. Figure 4 shows the flocculation kinetics of artificial clay-based tailings in treated and raw seawater obtained using the FBRM technique. It is important to recall that treated seawater has a reduced magnesium content according to the lime and sodium carbonate ratio used. Figure 5 shows the tailing settling rates measured when the flocculation test was interrupted at 20, 40, 60, and 80 s, which we call flocculation times. Every flocculation test began from scratch. In general, the kinetics led to large aggregates in the short term, but prolonged agitation produced fragmentation of the aggregates and thus a decrease in settling rates. This fragmentation was practically irreversible when the flocculant chains ere torn apart [29,30]. Agitation not only deteriorated the flocculation but also the quality of the seawater with respect to its magnesium content. When seawater treatment was performed with lime/sodium carbonate ratios below 50%, the flocculation kinetics were not much different from the kinetics in raw seawater, according to Figure 4.

Small aggregates, no more than ca. 120 microns, were formed, that rapidly disaggregated after brief stirring. When the treatment involved lime/sodium carbonate ratios over 50%, the amount of magnesium cations in the liquor decreased rapidly after the massive formation and separation of insoluble magnesium hydroxides. In the subsequent flocculation tests, large aggregates formed, which disaggregated more slowly, but significantly, with prolonged agitation. For example, when the content of lime in seawater was over 90%, the aggregates reached ca. 250 microns in a short time, and after stirring for 250 s, the average size reached ca. 150 microns. Such fragmentation as flocculation time increases was due to the sustained hydrodynamic shear and more frequent contact of magnesium complexes which invariably form at alkaline pH with tailings particles. These complexes are expected to eventually coat the tailings particles and flocculant chains, inhibiting their interactions. Therefore, flocculation improves with water treated with high lime/sodium carbonate ratios and deteriorates markedly with low ratios. In the latter case, the formation of magnesium complexes due to the strong alkalinity of the medium and the ion-pairing of magnesium ions and anionic groups in the flocculant suppresses the action of the flocculant in the same way as in raw seawater. In Figure 4, the size reached by the aggregates in treated and raw seawater can be determined when flocculation is interrupted after 20, 40, 60, and 80 s, and settling begins. Figure 5 summarizes the corresponding settling rate data.

The settling rates increased to more than acceptable values for the industry as the lime/sodium carbonate increased from 70 to 100% at all flocculation times, although the higher the flocculation time, the lower the settling rate. As the ratio increases toward 100%, almost all magnesium is precipitated in complexes, including magnesium hydroxide, and was removed prior to flocculation tests. However, the magnesium that does not precipitate during the treatment does so in the alkaline environment in which flocculation occurs. If the flocculation time is short (20 s in Figure 4 and Figure 5), the complexes do not interfere with the flocculation of the tailings particles; rather, they are incorporated into the process, forming large agglomerates that settle easily. However, if the flocculation under agitation lasts a long time (60 and 80 s in Figure 4 and Figure 5) before being interrupted, sedimentation is notoriously impeded due to fragmented aggregates and a degraded flocculant. The high concentration of calcium cations under high lime conditions does not appear to affect the flocculation of the tailings particles. Motta et al. [31] observed a synergistic effect between Ca^2+^ concentration and polyacrylamide dose leading to a beneficial effect on particle flocculation. However, there is no consensus; other works suggest that a high calcium concentration would affect the performance of flocculants due to coiling [32,33].

In seawater treated with lime/sodium carbonate ratios lower than 70%, the remaining magnesium content in the water is very high, and the sedimentation rates are unacceptably low, in the order of 1 m/h, only comparable to the rates obtained in raw seawater, as shown in Figure 5. The fate of the sedimentation in these cases and in raw seawater is decided in the flocculation stage regardless, of the flocculation time. In seawater with low lime (less than 50%), the concentration of magnesium that remains in solution after the treatment is over 920 mg/L, so it is ineffective for magnesium reduction, as shown in Figure 3. Then, in the strongly alkaline medium of flocculation, this remaining magnesium forms hydroxylated complexes that, being deposited on the particles and the flocculant chains, prevent their interaction. Additionally, the residual magnesium cations interact with the acrylate groups in the flocculant chains by ion-pairing, thus preventing the particle–flocculant interaction even more.

For Ramos et al. [23], the presence of magnesium precipitates distracts the flocculant from the tailings particles, generating selectivity problems. According to these authors, the magnesium precipitates effectively interact with the flocculant chains occupying their functional groups. In recent work, Quezada et al. [29] used molecular dynamics simulations to study the interaction between flocculants and brucite, the crystalline form of magnesium hydroxide. The interaction between the deprotonated oxygen of the acrylic group of the polymer and the oxygen on the brucite surface dominates. A minor but significant contribution is that of hydrogen bonds between the nitrogen of the acrylamide group and the oxygen on the surface of the brucite.

### 3.3. Effect of Flocculant Dose

**Figure 6:**
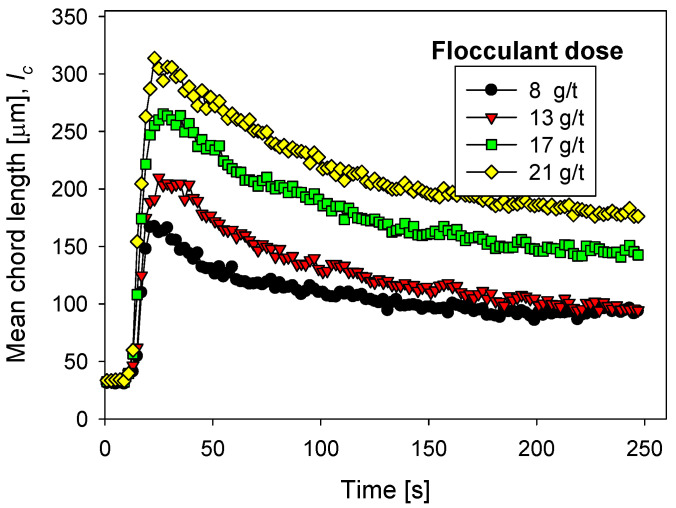
Clay-based tailings flocculation kinetics in seawater treated with lime and different flocculant doses. Quartz/kaolin = 80/20, pH 11, stirring rate 220 rpm.

**Figure 7:**
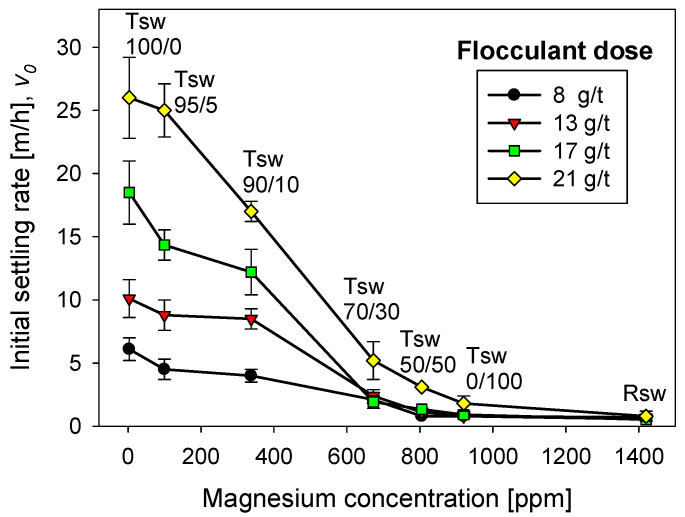
Effect of flocculant dose and seawater treatment on the settling rate of flocculated tailings. Quartz/kaolin = 80/20, pH 11, stirring rate 220 rpm, and flocculation time 20 s.

Figure 6 shows the flocculation kinetics of artificial clay-based tailings in seawater treated with lime and different flocculant doses. The FBRM technique provided the average size of the aggregates. Figure 7 shows the corresponding settling rates of the tailings. Settling rates were measured once the flocculation tests were interrupted after 20 s, that is, when the aggregates reached their largest size. Thus, each flocculation test began from scratch.

The flocculation kinetics in Figure 6 show a continuous growth of the tailing aggregates with increasing flocculant doses. For example, the size peaks at doses of 8 to 21 g/t ranged from 165 to 310 µm. It is also true that the larger the aggregates, the greater the fragmentation with the flocculation time. For example, at 21 g/t, after 250 s of flocculation, the aggregate size decreased from ca. 300 microns to less than 200 microns, a 66% relative decrease from the initial size. On the other hand, at 8 g/t, after 250 s of flocculation, the size decreased from ca. 175 microns to ca. 100 microns, that is, 57% of the initial size. Flocculation enhancement with flocculant dose also occurs in seawater treated with different lime/sodium carbonate ratios, but this amount was less than when the ratio was 100% lime. These data are not shown here but are available on request.

The sedimentation behavior of the tailings particles, once the agitation in the flocculation cell was stopped at 20 s, was similar for different doses of the flocculant (Figure 7). If the dose of flocculant was high (21 g/t) and the lime/sodium carbonate ratio was high (>90%), the settling rate was also high. As the lime/sodium carbonate ratio decreased, the settling rate also decreased, regardless of how high the flocculant dose was; this result was not different from that of raw seawater. If the flocculant dose was very low, such as 8 g/t, it did not matter much that the seawater treatment was only with lime, the settling rates were unacceptably low, almost as low as in seawater. Thus, seawater treatment for magnesium abatement and flocculant type/dose are critical for adequate solid–liquid separation. Both must be optimal to correctly control the rupture of the aggregates and the flocculant’s degradation, especially the flocculant’s interaction with the magnesium complexes formed in each seawater treatment and the ion pairing between the magnesium cations and the anionic sites of the flocculant chains. According to Ramos et al. [1], magnesium complexes monopolize the flocculant up to a critical dose. Above this dose, the flocculation of the mineral begins. The sedimentation data from seawater treated with a 70% lime/sodium carbonate ratio support this thesis. Settling was poor for flocculant doses of 8 to 17 g/t; however, at 21 g/t, a settling rate greater than 5 m/h was obtained. When the ratio was 100% lime and the flocculant dose was as low as 8 g/t, the settling rate was already acceptable, about 5 m/h, but doubling the flocculant dose quadrupled the settling rate. It is important to remember that Figure 5 corresponds to 20 s after stopping the flocculation test. This short time minimizes the interaction between the magnesium complexes and the flocculant chains.

### 3.4. Effect of the Kaolin Content

Clays are generally tricky to flocculate unless they are the flocculating agents [34]. Therefore, separating clays from water in large thickeners requires extremely well-defined conditions and well-designed flocculating polymers, especially if the ionic strength is high such as in raw seawater or partially desalinated or treated seawater.

**Figure 8:**
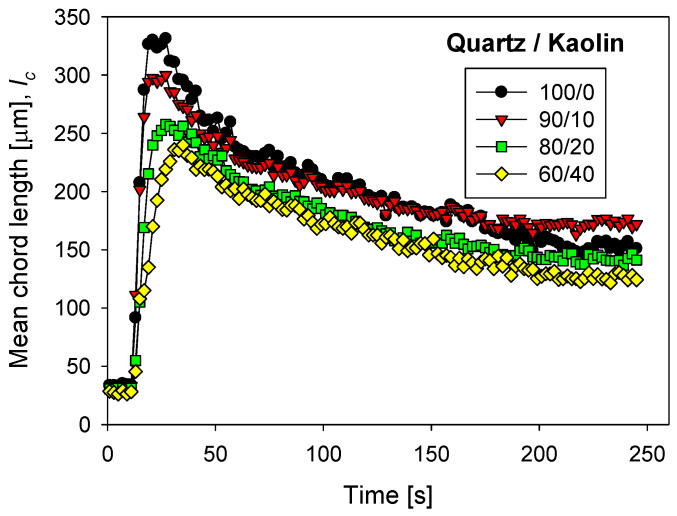
Clay-based tailings flocculation kinetics in seawater treated with lime with different quartz/kaolin ratios. pH 11, stirring rate 220 rpm, and flocculant dose 17 g/t.

Figure 8 shows the flocculation kinetics of tailings prepared with different quartz/kaolin ratios in seawater treated with lime to abate magnesium at a fixed flocculant dose of 17 g/t. The largest aggregates were formed in the absence of clay particles. The aggregates of quartz tailings reached a maximum size of 330 microns very quickly (ca. 20 s), while the aggregates of quartz and kaolin tailings in a 60/40 ratio reached less than 250 microns at longer times (ca. 40 s). Therefore, flocculation time requires special control. Large quartz aggregates break under prolonged shear, so the sedimentation operation should be carried out in short times, close to 20 s. Clay-rich aggregates are smaller. In this case, the settling operation must occur immediately after a sufficient particle–flocculant contact time, between 40 and 50 s. The flocculation kinetics curves for tailings in seawater treated with lime and sodium carbonate in different proportions are not shown here, but are available on request.

**Figure 9:**
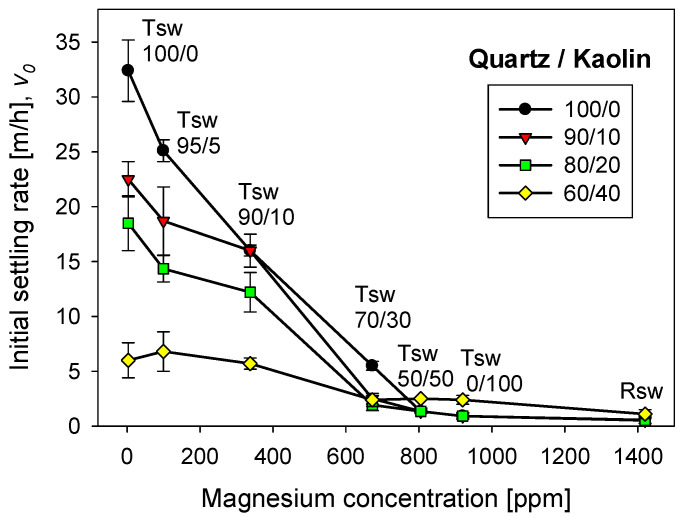
Effect of quartz/kaolin ratio and seawater treatment on the settling rate of flocculated tailings. pH 11, stirring rate 220 rpm, flocculant dose 17 g/t, and flocculation time 20 s.

Figure 9 shows the settling rates of tailings of quartz/kaolin for treated and raw seawater at a flocculant dose of 17 g/t. Settling rates were measured once the flocculation tests are interrupted after 20 s, that is, when the aggregates reached large sizes, although not exactly their maximum size, as was the case with the kaolin-rich tailings. The tests for each particle system were independent and began from scratch. Figure 9 shows that kaolin deteriorates sedimentation, reducing the settling rate, which was a known impact [16]. When the seawater treatment was with lime alone, practically all the magnesium formed insoluble complexes that were removed and discarded before the flocculation tests. The settling rate for quartz tailings exceeded 30 m/h, and the settling rate for quartz/kaolin tailings in a 60/40 ratio was only 5 m/h. These low settling rates were due to the smaller size of the aggregates in the presence of the kaolin particles, due to the fine size of the clay particles and their plate-like shape with a high specific surface that consumes flocculant. Interestingly, when using water treated with sodium carbonate only, almost all the magnesium remained in the ionic state in the solution. As a result, the settling rate was practically zero for tailings with kaolin content less than 20%, and 2 m/h for tailings with more than 40% kaolin. At first, it seems that these settling results are better for systems with higher clay content; however, when they are compared with results in raw seawater, it is seen that they are not different. The latter suggests that the treatment only with carbonate sodium (without lime), is unsuitable for systems with high clay content, from the point of view of sedimentation.

The thickening stages generally do not require sedimentation rates as high as those observed with seawater treated 100% with lime; in fact, discharge from underflows is hampered at high rates. Rates between 10 and 20 m/h can be considered acceptable in the copper mining industry. Therefore, removing all magnesium from seawater is unnecessary to achieve optimal sedimentation conditions. However, this depends on the clay content. The higher the kaolin content, the greater the amount of magnesium to abate.

### 3.5. Structure of Aggregates

**Figure 10:**
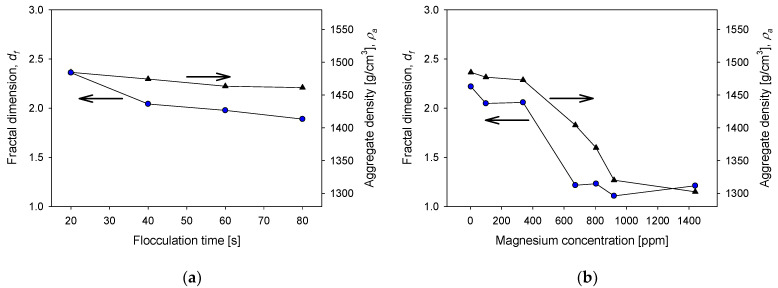

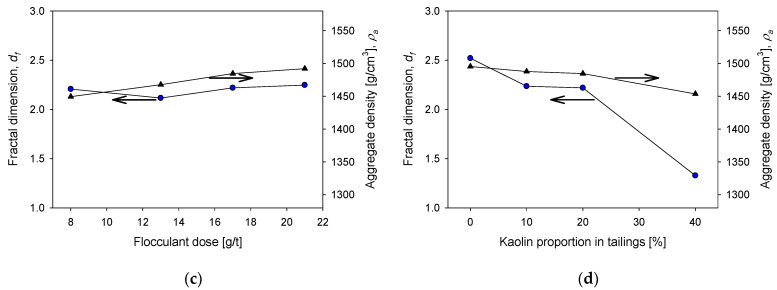
Fractal dimensions and density of aggregates as functions of (**a**) flocculation time (quartz/kaolin 80/20, flocculant dose 17 g/t, magnesium concentration 3 ppm), (**b**) magnesium concentration (quartz/kaolin ratio 80/20, flocculant dose 17 g/t, flocculation time 20 s), (**c**) flocculant dose (quartz/kaolin ratio 80/20, flocculation time 20 s, magnesium concentration 3 ppm), (**d**) and kaolin content in quartz tailings (flocculant dose 17 g/t, flocculation time 20 s, magnesium concentration 3 ppm). All tests were performed at pH 11.

The fractal dimension can be a handy indicator of the structural characteristics of tailings aggregates formed under different magnesium concentration conditions, which depend on the lime/sodium carbonate ratio, flocculant dosage, flocculation time, and kaolin content. The structural characteristics include compactness, resistance to hydrodynamic shear, the effectiveness of the flocculant at the applied dose, the effect of insoluble complexes formed during the process, and even permeability. In addition, depending on the type of system, flocculation conditions define the structure of the aggregates and the settling behavior [28,35]. Figure 10 shows the fractal dimensions and densities of aggregates of quartz and kaolin tailings at various conditions. Figure 10a shows that the fractal dimension and density of the aggregates change little if the flocculation time is extended. Thus, the size of the aggregates decreases with flocculation time, but their structure and density remain relatively constant. For example, the fractal dimension starts with a value of 2.36 at 20 s of flocculation and ends at 2.21 after 80 s of flocculation. The density begins at a value of at 1480 g/cm^3^ at 20 s and ends at 1410 g/cm^3^ at 80 s. These results are in agreement with those of Quezada et al. [36], who observed that aggregates from mine tailings flocculated with anionic polyacrylamides maintain their structure when the shear rate is less than 200 s^−1^. The effect of the magnesium content is different when seawater is treated with varying proportions of lime and sodium carbonate. Figure 10b shows that the fractal dimension of the aggregates at 400 ppm magnesium were ca. 2.3 but at a high magnesium concentration at the limit of raw seawater, the fractal dimension reached values close to 1. The implications for the water recovery operation are dramatic: sedimentation is very slow because the aggregates are structurally very loose and light, and water clarification may require prohibitive times. The aggregates are so light that the density of the aggregates decreases from 1480 g/cm^3^ in liquor with 400 ppm magnesium to 1320 g/cm^3^ in raw seawater. Regarding the dose of the flocculant, Figure 10c shows that the higher the dose, the greater the fractal dimension, although the increase is slight, from 2.2 to 8 g/t to 2.4 to 21 g/t. This result is consistent with the results of a previous study by Jeldres et al. [36]. In contrast, the density of the aggregates remained relatively constant at 1450 g/cm3 in the dose range used. Finally, Figure 10d shows the impact of the clay content of the tailings on the fractal dimension and the density of the aggregates that are formed, both of which decrease. The fractal dimension decreased slightly, maintaining values between 2.4 without kaolin and 2.3 with 40% kaolin. The density of the aggregates decreased dramatically from 1500 g/cm^3^ without kaolin to 1330 g/cm^3^ with 40% kaolin. Here, the difficulty for the water recovery operation is the lightness of the aggregates in tailings with high clay content, the clarification times in the latter case would also be prohibitive.

## 4. Conclusions

Seawater treated with different lime and sodium carbonate proportions was used to improve the flocculation and sedimentation of clay-based tailings under highly alkaline conditions. The precipitated solids, mainly magnesium and some calcium, were separated from the water by vacuum filtration. When operating with raw seawater at pH 11, solid magnesium complexes impair the selectivity of the flocculating polymer, drastically reducing its performance. However, promising results were obtained when using magnesium control-treated seawater, including a significant increase in the sedimentation rate caused by structural changes in the aggregates, including size, fractal dimension, and density. A low fractal dimension revealed open and porous structures, intensifying at high magnesium concentrations and clay contents. If, in addition, the density of the aggregates was low, then the sedimentation of the tailings and the clarification of water were of little practical use. The results suggest that both the flocculation and the sedimentation of low-clay content tailings occur with industrially attractive yields even in the presence of magnesium. However, the flocculation and sedimentation of high-clay tailings requires a significant lowering of the magnesium concentration to be of industrial interest. The latter can be achieved by using seawater treated with lime.

## Figures and Tables

**Table 1:** Ionic concentration of seawater and analytical methods.

Ion	Concentration [g/L]	Analytical Method
Na^+^	10.8	Atomic absorption spectrometry
Mg^+^	1.42	Atomic absorption spectrometry
Ca^2+^	0.42	Atomic absorption spectrometry
K^+^	0.39	Atomic absorption spectrometry
Cl^−^	18.9	Argentometry
HCO_3_^−^	0.14	Volumetric acid-base titration

**Table 2:** Ionic concentration of seawater and analytical methods.

Product	Solubility Product (Ksp)
Mg(OH)_2_	5.61 × 10^−12^
MgCO_3_	6.82 × 10^−6^
Ca(OH)_2_	5.02 × 10^−6^
CaCO_3_	3.36 × 10^−9^

## Data Availability

The data presented in this study are available on request from the corresponding author.
